# 

**Published:** 1845-04

**Authors:** 


### BIBLIOGRAPHICAL RECORD.

1. Treatise on Inflammation as a Process of Anormal Nutrition. By JOHN HUGHES BENNET, M.D.

2. The Anatomy and Physiology of Expression, as connected with the Fine Arts. By Sir CHARLES BELL, K.H.

3. Researches and Observations on the Causes of Scrofulous Diseases. By J. G. LUGOL. Translated by Dr. RANKING.

4. A Manual of Elementary Chemistry, Theoretical and Practical. By G. FOWNES, Ph. D.

5. The Principles of Human Physiology, with their chief Application to Pathology, Hygiene and Forensic Medicine, especially designed for the Use of Students. By W. B. CARPENTER, M.D. 2nd edition.

6. A Treatise on the Use of the Sympathetic Nerve and its Ganglions. By T. B. PROCTOR, M.D. Illustrated with Drawing.

7. An Apology for the Nerves, or their Influence and Importance in Health and Disease. By Sir G. LEFEVRE, M.D.

8. Elements of Anatomy, intended as a Text-Book for Students. By A. J. LIZARS, M.D.

9. Urinary Deposits; their Diagnosis, Pathology, and Therapeutical Indications. By GOLDING BIRD, A.M. M.D.

10. The Medical Remembrancer, or Book of Emergencies. By E. B. L. SHAW, M.R.C.S.

11. The Medical Report of the Case of Miss H. M—. By T. M. GREENHOW, F.R.C.S.

12. Toxicological Chart of the various Poisons, with their Symptoms, Treatment, and Modes of Detection. By W. STOWE, M.R.C.S. 10th Edit.

13. Scrofula, its Nature, Causes, and Treatment. By W. TYLER SMITH.

14. The Natural History of Animals, being the Substance of Three Courses of Lectures, delivered before the Royal Institution of Great Britain. By THOS. RYMER JONES, F.R.S. F.Z.S.

15. A Report of the Obstetric Practice of the University College Hospital,

London. By Edward W. Murphy, A.M. M.D.

16. An Explanation of the real Process of Spontaneous Evolution of the Fœtus. By John C. Douglas, M.D. Third Edition.

17. A Retrospect of Practical Medicine and Surgery. By W. Braithwaite. Vol. X. July to Dec. 1844.

18. Outlines of Chemistry, for the Use of Students. By Wm. Gregory, M.D. Part I. Inorganic Chemistry.

19. The Philosophy of the Moving Powers of the Blood. By G. Calvert Holland, M.D.

20. A Thermometrical Table on the Scales of Fahrenheit, Centigrade, and Reaumur; comprising the most remarkable Phenomena, Chemical and Physiological, connected with Temperature. By Alfred S. Taylor, Lecturer on Chemistry in Guy's Hospital.

21. Practical Observations and Suggestions in Medicine. By Marshall Hall, M.D. F.R.S.

22. The Northern Journal of Medicine, a Monthly Survey of the Progress of Medical Knowledge at Home and Abroad. February, 1845.

23. The Anatomist's Vade Mecum; a System of Human Anatomy. By Erasmus Wilson. Third Edition.

24. The Chemistry of Vegetable and Animal Physiology. By D. G. J. Mülder, Professor of Chemistry in the University of Utrecht. Translated from the Dutch, by Dr. Fromberg.

25. Consumption, its Curability established by Reference to numerous Cases in the Author's Practice. By D. Cronin, M.D. Gissen.

26. General Report of the Royal Hospitals of Bridewell and Bethlem, and of the House of Occupations, for the Year 1844.

27. The American Journal of Insanity. Edited by the Officers of the New York State Lunatic Asylum. Utica, January 1845.

28. Anatomical and Pathological Observations. By John and Harry D. S. Goodsir.

29. The American Journal of Medical Sciences. Edited by Isaac Hays, M.D.

30. The Medical Examiner and Record of Medical Science, No. 20 to 26. (Philadelphia.) Edited by Robert M. Huston, M.D.

31. Medical Education; being a Lecture delivered at King's College, London. By J. Forbes Royle, M.D. &c.

32. Medicina Gymnastica, or Therapeutic Manipulations: a short Treatise on this Science, as practised at the Royal Institution at Stockholm. By Charles Ehrenhoff.

33. Rural Economy, in its Relations with Chemistry, Physics, and Meteorology; or an Application of the Principles of Chemistry and Physiology to the Details of Practical Farming. By T. B. Boussingault. Translated, with an Introduction and Notes, by George Law, Agriculturist.

34. The Anatomy of Sleep, or the Art of procuring Sound and refreshing Slumber at will. By Edward Binns, M.D. 2nd edition.

35. Essay upon Cretinism and Goitre. By Edward Wells, M.D.

36. The Actual Process of Nutrition and Inflammation in the Living Structure, demonstrated by the Microscope. By William Addison, F.L.S.

37. Mesmerism true — Mesmerism false: a Critical Examination of the Facts, Claims, and Pretensions of Animal Magnetism. Edited by John Forbes, M.D. F.R.S. With an Appendix, containing a Report of two Exhibitions by Alexis.

38. An Address to the Middle and Working Classes on the Causes and Prevention of the excessive Sickness and Mortality prevalent in large Towns. By Wm. Strange, M.D., Edinb.

39. Sir James Eyre on Oxyd of Silver.

40. Dr. Coley on the Cæsarian Operation.

